# The genetic control of leaf and petal allometric variations in *Arabidopsis thaliana*

**DOI:** 10.1186/s12870-020-02758-w

**Published:** 2020-12-07

**Authors:** Xin Li, Yaohua Zhang, Suxin Yang, Chunxia Wu, Qun Shao, Xianzhong Feng

**Affiliations:** 1 CAS Key Laboratory of Soybean Molecular Design Breeding, Northeast Institute of Geography and Agroecology, Chinese Academy of Sciences, Changchun, 130102 China; 2 Shandong Provincial Key Laboratory of Plant Stress, College of Life Sciences, Shandong Normal University, Jinan, 250014 China

**Keywords:** *Arabidopsis thaliana*, Leaf and petal, Allometric variation, QTL mapping, Multiparent advanced generation intercross lines

## Abstract

**Background:**

Organ shape and size covariation (allometry) factors are essential concepts for the study of evolution and development. Although ample research has been conducted on organ shape and size, little research has considered the correlated variation of these two traits and quantitatively measured the variation in a common framework. The genetic basis of allometry variation in a single organ or among different organs is also relatively unknown.

**Results:**

A principal component analysis (PCA) of organ landmarks and outlines was conducted and used to quantitatively capture shape and size variation in leaves and petals of multiparent advanced generation intercross (MAGIC) populations of *Arabidopsis thaliana*. The PCA indicated that size variation was a major component of allometry variation and revealed negatively correlated changes in leaf and petal size. After quantitative trait loci (QTL) mapping, five QTLs for the fourth leaf, 11 QTLs for the seventh leaf, and 12 QTLs for petal size and shape were identified. These QTLs were not identical to those previously identified, with the exception of the ER locus. The allometry model was also used to measure the leaf and petal allometry covariation to investigate the evolution and genetic coordination between homologous organs. In total, 12 QTLs were identified in association with the fourth leaf and petal allometry covariation, and eight QTLs were identified to be associated with the seventh leaf and petal allometry covariation. In these QTL confidence regions, there were important genes associated with cell proliferation and expansion with alleles unique to the maximal effects accession. In addition, the QTLs associated with life-history traits, such as days to bolting, stem length, and rosette leaf number, which were highly coordinated with climate change and local adaption, were QTL mapped and showed an overlap with leaf and petal allometry, which explained the genetic basis for their correlation.

**Conclusions:**

This study explored the genetic basis for leaf and petal allometry and their interaction, which may provide important information for investigating the correlated variation and evolution of organ shape and size in *Arabidopsis*.

**Supplementary Information:**

The online version contains supplementary material available at 10.1186/s12870-020-02758-w.

## Background

Organ morphology is determined by organ shape and size, and coordinated variation in shape and size is a major component of natural diversity. Allometry refers to the size-related changes in morphological traits and can be used to describe the correlated variation in shape and size that can occur within one type of organ or can involve the relative proportions of different organs [1–3]. The homologous organs, leaves and petals, may share a basic developmental control machinery [4–6], and the correlations between their organ size and shape are possibly regulated by some allometry factors. Even closely related species can still show very different allometries, possibly due to the correlations resulting from selection [7, 8] and developmental constraints [9]. The genetic and evolutionary basis for allometric variation is integral to our understanding of plant development. However, these are poorly understood.

To address this question, some quantitative genetic frameworks have been built up to quantify organ shape and size [1, 3, 10–12]. With these frameworks both the size and shape differences are measured and compared to resolve the allometry variance between different organs. A point and outline approach was first amplified to quantify allometric variation within the leaves of the snapdragon (*Antirrhinum*) species [3]. This method captures allometric variation directly without resulting in shape and size separation and achieves the incorporation of different types of organs within the same framework. It has been applied to quantify the allometric variation of leaves and petals in *Antirrhinum* and *Arabidopsis*, to investigate the genetic basis of organ size and shape developmental mechanism [1, 10, 11].

*Arabidopsis thaliana* is an ideal organism for the study of natural variation in leaf and petal shape and size because there are extensive variations among worldwide accessions for both of these traits and for many life-history traits [13–15]. Leaves and petals have an advantage, as both their shapes and sizes can be readily captured for an initial approximation by a two-dimensional (2D) outline. Previous studies specifically featured a QTL analysis of leaf and petal shape and size in *Arabidopsis thaliana*. Recombinant inbred lines (RILs) from a *Ler-0* × *Col-4* cross identified a total of 16 and 13 QTL-harbouring, naturally occurring alleles that contributed to natural variations in the architecture of juvenile and adult leaves, respectively [16]. In the *Ler* × *Cvi* RIL population, eight QTLs for petal traits and three QTLs for leaf traits were identified [17]. Abraham et al. [18] found 23 QTLs for variation in petal length, width, area, and shape in two RIL populations (*Col-0* × *Est-1* and *Ler-0* × *Col-4*). In addition, many factors controlling leaf and petal shape and size have been identified and have been shown to be regulated by hormonal signals, transcription factors and miRNAs during leaf and petal development, and recent findings have highlighted the contribution of mechanical signals to leaf and petal growth [19–21]. However, neither these QTLs nor the factors identified could capture the allometry variation of leaves and petals due to the limitation of the common measures in capturing the shape variation fully and in integrating the analysis of shape and size [22].

In this study, we investigated the genetic basis of natural allometry variation in leaves and petals using a set of RILs of *Arabidopsis thaliana* that were derived from MAGIC lines, which were constructed by 19 founder accessions [14]. Multiparent lines are better for addressing genetic correlations due to the larger number of alleles and recombination events, which allows for mapping to smaller intervals [14]. In addition, the larger number of alleles improves the ability to determine whether the distributions of allelic effects are compatible with pleiotropy. Moreover, we used a quantitative approach based on a PCA of landmark positions to define allometric spaces that captured variation in shape and size [1, 3, 10, 11], which was treated collectively to allow allometric relationships to be defined.

## Results

### Allometry models of leaves and petals in MAGIC lines

**Fig. 1:**
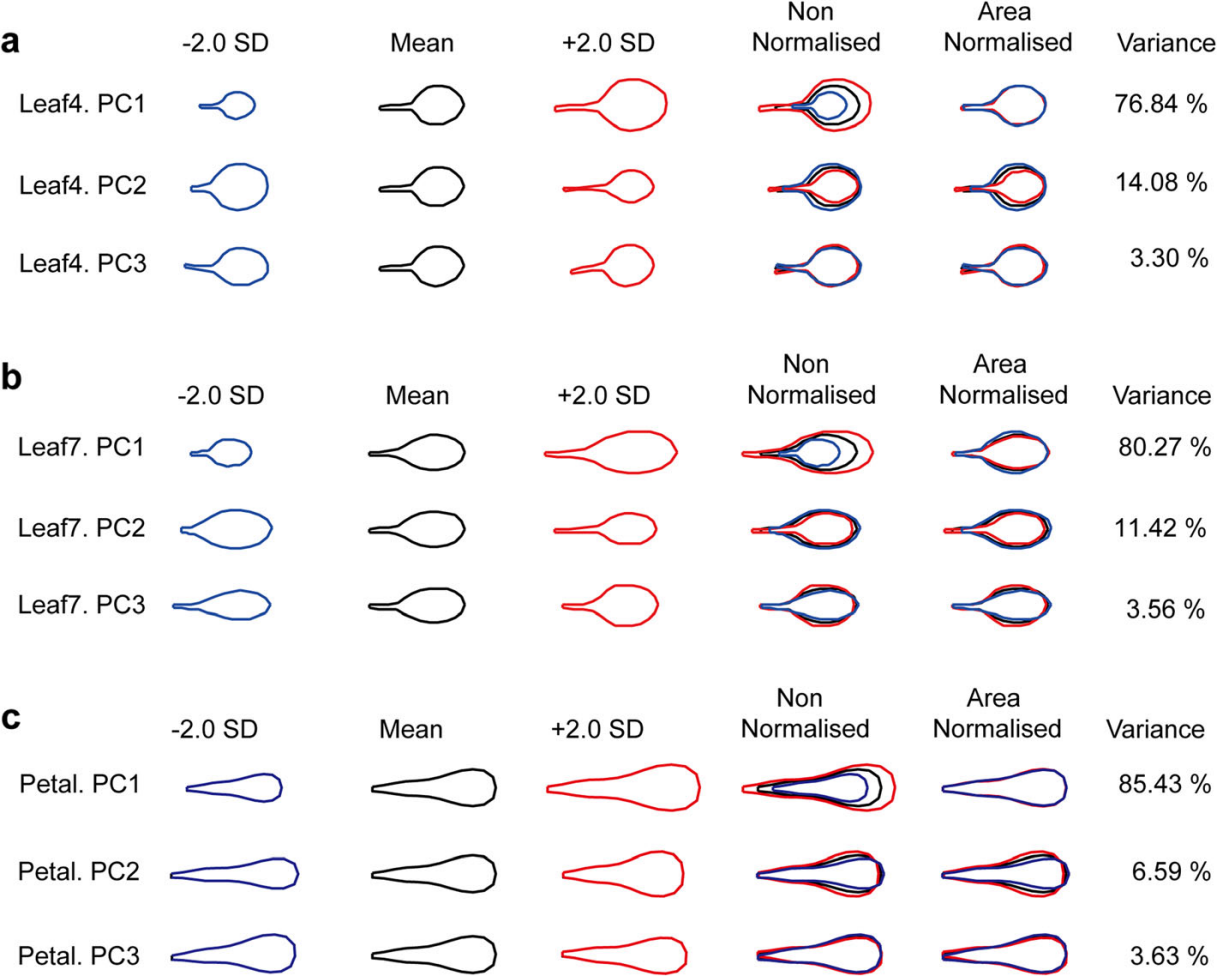
Leaf and petal allometry models for MAGIC lines. Allometry models describe variations in the shape and size of the fourth leaves (**a**), seventh leaves (**b**) and petals (**c**) in terms of principal components (PCs). The mean effects in each PC for all samples are represented by black outlines. The blue outlines correspond to the decrease by two standard deviations from the mean, and the red outlines correspond to the increase by two standard deviations. The overlaid outlines with area non-normalized illustrate the effects of each PC on organ shape and size, and the overlaid outlines adjusting to the same area (area-normalized) illustrate the effects on organ shape. The proportion of the variance within the group captured by each PC to the total variance is given as a percentage

To detect the shape and size variation of leaves and petals within *Arabidopsis thaliana*, an allometric method based on a PCA of organ landmarks and outlines was used to quantify this trait. Leaf4, Leaf7 and petals from MAGIC lines were modelled and generated a separate data set. PCA was applied and the resulting principal components (PCs) were ranked according to the proportions of the total variance that each of them described (Fig. 1, Supplementary Figure 1).

In Leaf4, the PCA revealed that 90.92% of the variance in organ shape and size was attributed to two PCs (Fig. 1a). Leaf4.PC1 accounted for 76.84% of the total variance and affected the leaf size. Higher PC1 values corresponded to larger leaves, whereas lower values yielded smaller leaves. PC2 accounted for 14.08% of the variance and reflected the variations of leaf shape and petiole length. Plants with higher values of PC2 exhibited more elongated leaves with longer petioles, while lower with more rounded leaves and shorter petioles. PC3 explained 3.30% of the variance and displayed the degree of petiole twisting when the leaves were flattened, but its values were not significantly different between genotypes.

In Leaf7, the PCA revealed that 95.25% of the variance in organ shape and size could be attributed to three PCs (Fig. 1b). In this model, Leaf7.PC1 caused 80.27% of the total variance, which mostly influenced leaf size, but also had a minor effect on shape. Higher PC1 values corresponded to larger, more elongated leaves, whereas lower values yielded smaller and more rounded leaves. PC2 was responsible for 11.42% of the variance and mostly arranged the steepness of the transition from petiole to blade. Higher PC2 values yielded longer petioles with a steep transition, and lower values yielded shorter petioles with a very gradual transition. PC3 accounted for 3.56% of the variance and affected mainly the shape. Lower values of PC3 yielded more elongated and narrower leaves, whereas higher values of PC3 yielded more rounded and wider leaves.

In petals, the PCA revealed that 92.02% of the variance in organ shape and size could be attributed to two PCs (Fig. 1c). The PC1 accounted for 85.43% of the total variance and affected petal size. Higher PC1 values corresponded to larger petals, whereas lower values yielded smaller petals. PC2 accounted for 6.59% of the variance and affected mainly the shape. Low values of PC2 yielded elongated petals with a narrower shape, and high values of PC2 yielded rounded petals with a wider shape. PC3 accounted for 3.63% of the variance and was reflected in petal twisting when the petals were flattened. It was excluded from further analysis, because we could not detect significant differences in different genotypes.

The quantitative leaf and petal variations were captured by allometric models as PC values among the MAGIC lines (Table 1, Supplementary Figure 2). Extensive phenotypic variation was observed for all traits measured among the MAGIC lines, and the relative genetic contribution was estimated by broad-sense heritability (*H*^*2*^). The range of the *H*^*2*^ is from 0.62 to 0.87, which suggested the phenotypic variation among different lines was more attributed to the genetic component.

**Table 1:** Phenotypic variation among MAGIC lines for leaf and petal allometry models

Trait	Min	Max	Mean ± SD	*H*^*2*^
Leaf4.PC1	−3.24	2.70	−0.08 ± 0.99	0.83
Leaf4.PC2	−2.22	2.13	0.13 ± 0.90	0.67
Leaf7.PC1	−3.22	2.56	−0.14 ± 0.99	0.87
Leaf7.PC2	−2.18	1.96	0.10 ± 0.87	0.77
Leaf7.PC3	−2.66	2.39	−0.02 ± 0.87	0.62
Petal.PC1	−2.67	2.07	0.09 ± 0.96	0.83
Petal.PC2	−2.38	2.44	−0.14 ± 0.85	0.74

Minimum (Min) and maximum (Max) phenotypic values for each trait, as well as the phenotypic means plus or minus their standard deviation (SD) and their broad-sense heritability (*H*^*2*^), are shown

**Fig. 2:**
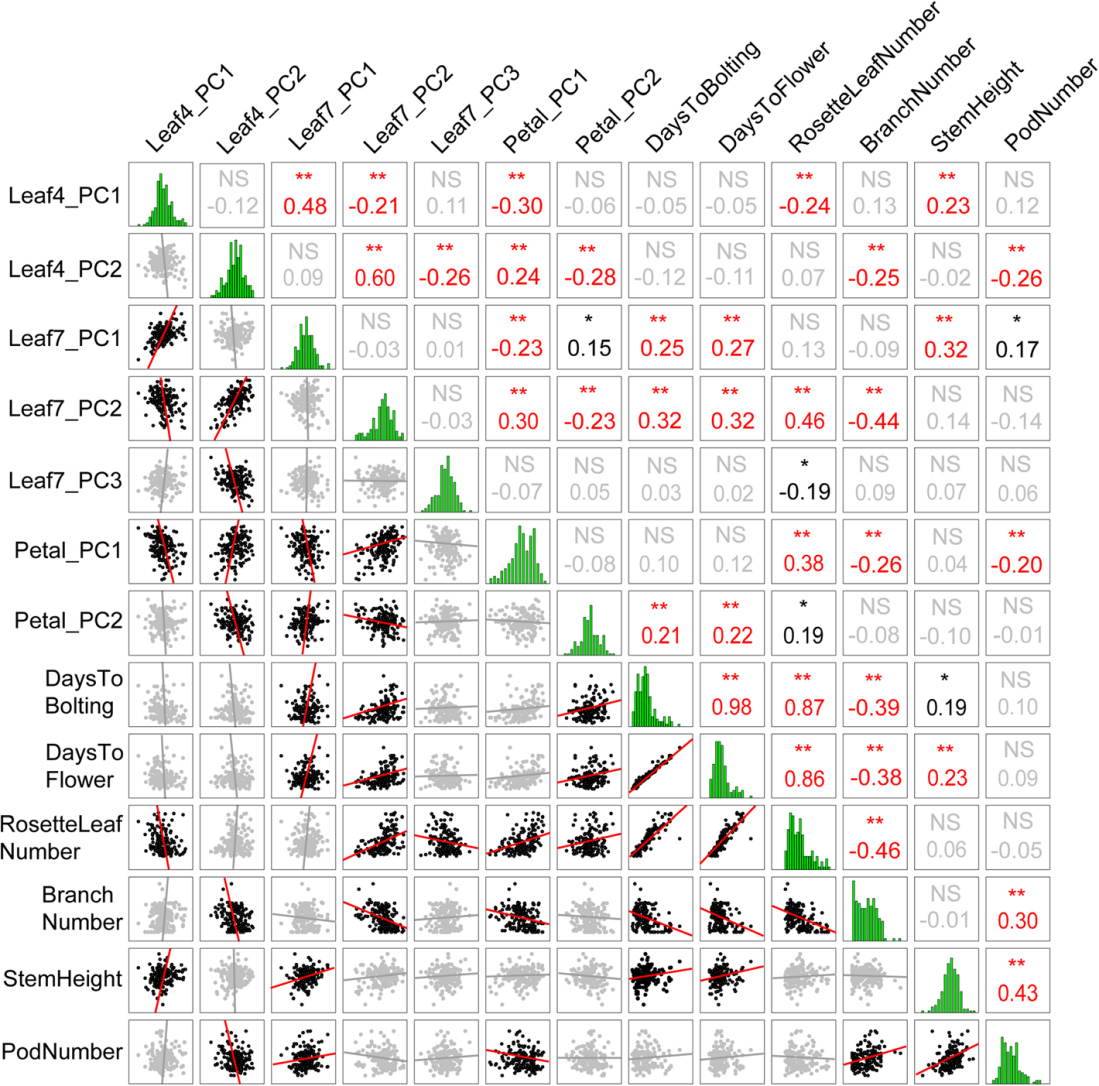
Pairwise Pearson’s correlations between the traits measured. All traits measured showed a normal distribution and Pearson’s correlations for leaf and petal allometry, and life history traits in MAGIC lines were significantly correlated in red (*P* < 0.01) and dark (*P* < 0.05), while they were not significant in grey. NS, not significant; **, correlation is significant at the 0.01 level (two-tailed); *, correlation is significant at the 0.05 level (two-tailed)

A correlation analysis between shape and size was also performed, and a number of significant pairwise correlations were observed (Fig. 2). Leaf4.PC1 was significantly positively correlated with Leaf7.PC1, which represented the leaf size. Leaf4.PC2 was significantly correlated with Leaf7.PC2 and leaf7.PC3, which represented the leaf shape. Moreover, leaf shape and size showed significant correlations with petals. Petal.PC1 was significantly correlated with Leaf4.PC1 and Leaf7.PC1, which showed a negative size correlation between leaves and petals. Furthermore, both Leaf4.PC2 and Leaf7.PC2 were significantly positively correlated with Petal.PC1 and negatively correlated with Petal.PC2. The correlation between the leaf and petal allometry model indicated the genetic dependency and evolution correlation controlling leaf and petal allometry. Besides, a pairwise correlation analysis was performed between the life history traits and the leaf and petal allometry model (Fig. 2). Leaf4.PC1 was correlated with rosette leaf number and stem height; additionally, Leaf4.PC2 was highly correlated with branch number and pod number; Leaf7.PC1 was correlated with days to bolting, days to flower and stem height; Leaf7.PC2 was highly positively correlated with days to bolting, days to flower, rosette leaf number, and branch number; Petal.PC1 was correlated with rosette leaf number and branch number; and Petal.PC2 was correlated with days to bolting and days to flower.

### QTLs accounted for leaf and petal allometry

To examine the genetic basis for shape and size variation of leaves and petals along the PCs in the MAGIC lines, we treated each PC as a quantitative trait, whose variation frequency showed a normal distribution (Fig. 2, Supplementary Figure 3) for QTL mapping. In QTL mapping of the MAGIC lines, the PCs for the leaf and petal allometry model and 1260 SNP markers among the 19 founder ecotypes were used. We then calculated a series of QTLs associated with the variance of leaf and petal shape and size (Fig. 5, Supplementary Table 2, Supplementary Figures 4, 5 and 6). In the leaf model, the QTL analysis for Leaf4.PC1 identified four QTLs located on chromosomes 1 and 3 and one QTL located on chromosome 2 for Leaf4.PC2. For Leaf7.PC1, five QTLs were observed on chromosome 3, one QTL was located on chromosome 2 for Leaf7.PC2, and four QTLs were located on chromosomes 1 and 2 for Leaf7.PC3. In the petal model, three QTLs were identified on chromosomes 1 and 4 in Petal.PC1, and nine QTLs were identified on chromosomes 1, 2, 3, and 5 in Petal.PC2.

After comparing the positions for all the QTLs identified, there was some QTL overlapping in the leaf and petal allometry model. The QTLs for PC2 of the leaf (Leaf4.PC2: LF4_2.1, Leaf7.PC2: LF7_2.1) and petal (Petal.PC2: PE_2.5) on chromosome 2 (~ 11 Mb) overlapped, and the alleles from the *Ler*-0 accession formed the most rounded leaves and petals with the widest shape (Supplementary Table 3). This QTL likely stemmed from the mutation of *ERECTA*, which is known to affect fruit length and is due to the allele from the *Ler-0* accession [18]. With the exception of the ER locus for leaf and petal shape, the QTLs LF7_1.1, LF7_1.2, LF7_1.3, LF7_1.4, and LF7_1.5 for Leaf7.PC1 on chromosome 3 overlapped with QTL PE_2.6 for Petal.PC2. Moreover, the QTLs LF7_1.3, LF7_1.4, and LF7_1.5 also overlapped with QTL PE_2.7 for Petal. PC2, whereas these QTLs all showed an uncorrelated allelic effects distribution (Supplementary Table 3). For the fourth and seventh leaves, except for the overlapping ER locus (Leaf4.PC2: LF4_2.1, Leaf7.PC2: LF7_2.1) for PC2 described above, the QTLs LF4_1.3 and LF4_1.4 for Leaf4.PC1 overlapped with QTL LF7_1.6 for Leaf7.PC1 on chromosome 3 and showed the same allelic effects distribution with a maximum value in the *Mt-0* accession and a minimum value in the *Can-0* accession (Supplementary Table 3). The overlapping QTLs might have explained the phenotypic correlation and indicated the correlated genetic modules for leaf and petal allometry in evolution.

### Candidate genes for leaf and petal allometry

The genes that explain natural variations in leaf and petal allometry have remained largely unknown. To identify possible candidate genes, we searched for genes containing nonsynonymous SNPs unique to accession according to PC distribution among these accession alleles (Supplementary Table 3). Based on the resequencing and reannotation of the 19 parental accessions [23], we identified candidate genes with unique alleles referring to the maximal effects accession in the 95% confidence region (Supplementary Table 4). In the Leaf4 allometry model, the auxin receptor *TIR1*, brassinolide signalling regulator *BSL3*, and *TIR1*, contributing to flowering time repression, had allelic variations in the coding sequence unique to the accession. In the Leaf7 allometry model, hormonal-related genes, such as *SUA* (a suppressor of *abi3–5)*, *ARGOS*, serine/threonine-protein kinase *PID2*, *BRI1 suppressor 1 (BSU1)-like 3*, and *ABI4* genes, had allelic variations in the coding sequence. Moreover, the flower time regulators *ELF3* and *ELF4*, the receptor kinase *ERECTA*, cell wall modification-related genes and some transcription factors conferred allelic variations unique to the maximal effects accession.

In the petal allometry model, 23 genes were identified with variations unique to the accession. Among these genes, *PTL* in Petal.PC2 encodes a trihelix transcription factor whose expression is limited to the margins of floral and vegetative organs. It is involved in limiting lateral growth of organs, and recessive mutations have been found to be defective in organ initiation and orientation in the second whorl [24]. The *OFP13* in Petal.PC2 encodes a member of the plant-specific OVATE family of proteins. Members of this family have been shown to bind to KNOX and BELL-like TALE class homeodomain proteins and function as transcriptional repressors that suppress cell elongation [25]. The *SEU* in Petal.PC1 encodes a transcriptional coregulator that coordinates with LEUNIG to regulate petal shape by controlling blade cell number and vasculature development within the petal [26]. Other genes, including the cell cyclin-related protein Cyclin A1;1, the protein kinase, the CYP family protein, the photoperiod-associated ELF6, and the transcription-related genes with nonsynonymous SNPs also contribute to petal PCs. The identified QTLs and candidate genes provided us with a valuable reference for insight into leaf and petal allometry.

### The genetic basis for leaf and petal covariation in allometry models

To examine the genetic basis for shape and size covariation between leaves and petals, the leaf and petal modelled data sets obtained above were combined to create Leaf4-Petal and Leaf7-Petal data sets, which allowed overall trends to be identified. To ensure equal weighting of the data from different organs, a constant factor was multiplied to the organ size for all plants as previous study [1]. The major correlated variations were detected by PCA analysis with both Leaf4-Petal and Leaf7-Petal data sets.

**Fig. 3:**
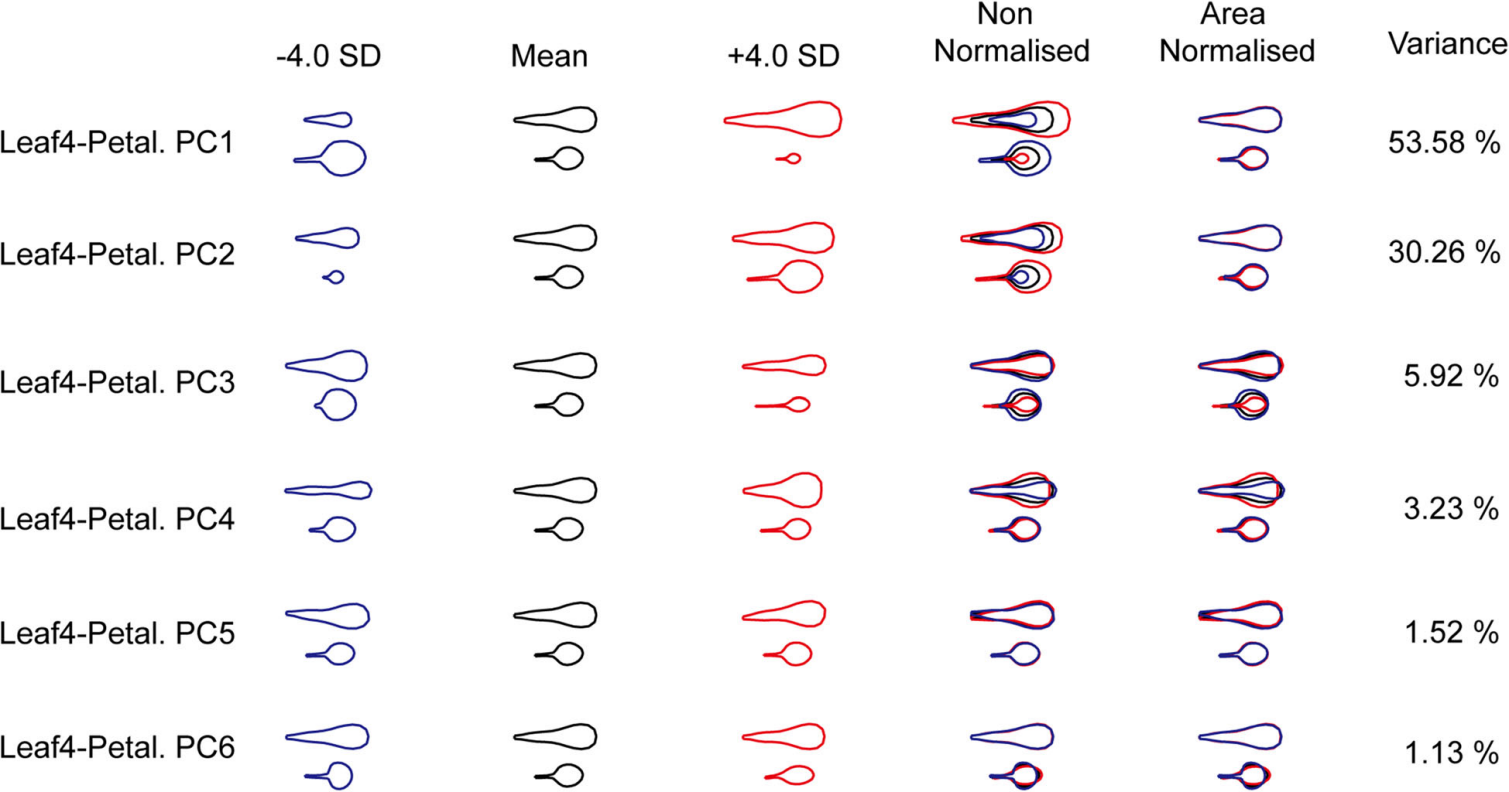
An integrated organ allometry model for the fourth leaves and petals. The effects on the leaf and petal outlines with decrease or increase in each PC by four standard deviations from the mean (black outlines) for all samples are represented by blue and red outlines respectively. The overlaid outlines illustrate the effects of each PC on organ shape and size covariation (area non-normalized) and organ shape covariation (area-normalized) separately. The proportion of the variance within the group captured by each PC to the total variance is given as a percentage

In the Leaf4-Petal model, PC1 accounted for 53.58% of the total variance, representing the negative size covariation between Leaf4 and petals. The higher the PC1 value, the larger the petal size, and the smaller the fourth leaf size. PC2 accounted for 30.26% of the total variance, representing the positive size covariation between the fourth leaves and petals. The higher the PC2 value, the larger the petal and leaf size. PC3 accounted for 5.92% of the total variance representing the positive shape (mainly in width) covariation between the fourth leaves and the petals. The higher the PC3 value, the more rounded the leaves and petals, and the shorter the petioles. PC4 accounted for 3.23% of the total variance representing the negative shape (mainly in width) covariation between the fourth leaves and the petals. The higher the value, the narrower the leaves, the longer the petioles, and the more rounded the petals were. The other PCs represented only one organ shape or size variance, so they were not considered for further analysis (Fig. 3).

After QTL mapping in the MAGIC lines for the Leaf4-Petal model, three significant QTLs for PC1, one significant QTL for PC2, two significant QTLs for PC3, and six significant QTLs for PC4 were identified (Fig. 5, Supplementary Table 5, Supplementary Figure 7). In each QTL, the candidate genes containing nonsynonymous SNPs unique to the maximal effects accession in the 95% confidence region were identified (Supplementary Tables 6 and 7). In PC1, there were five genes with the unique maximal effects accession allele, including the cell-proliferation-related genes, such as *ARGOS*, *LOM2*, and *EXPB5*. In PC3, which represented the shape (mainly in width) covariation, four genes were identified: *ARGOS*, *FRS3*, *BSL3*, and *extensin proline-rich1*. In PC4, representing the negative shape (mainly in width) covariation, there were also four genes containing the unique accession allele. Among these genes, the *CYCD2;1* gene acting on the G1 phase of the cell cycle to control the cell division rate in both the shoot and root meristems had an allele unique to the *Hi-0* accession, and the *PRX53* gene influencing cell elongation had an allele unique to the *Po-0* accession.

**Fig. 4:**
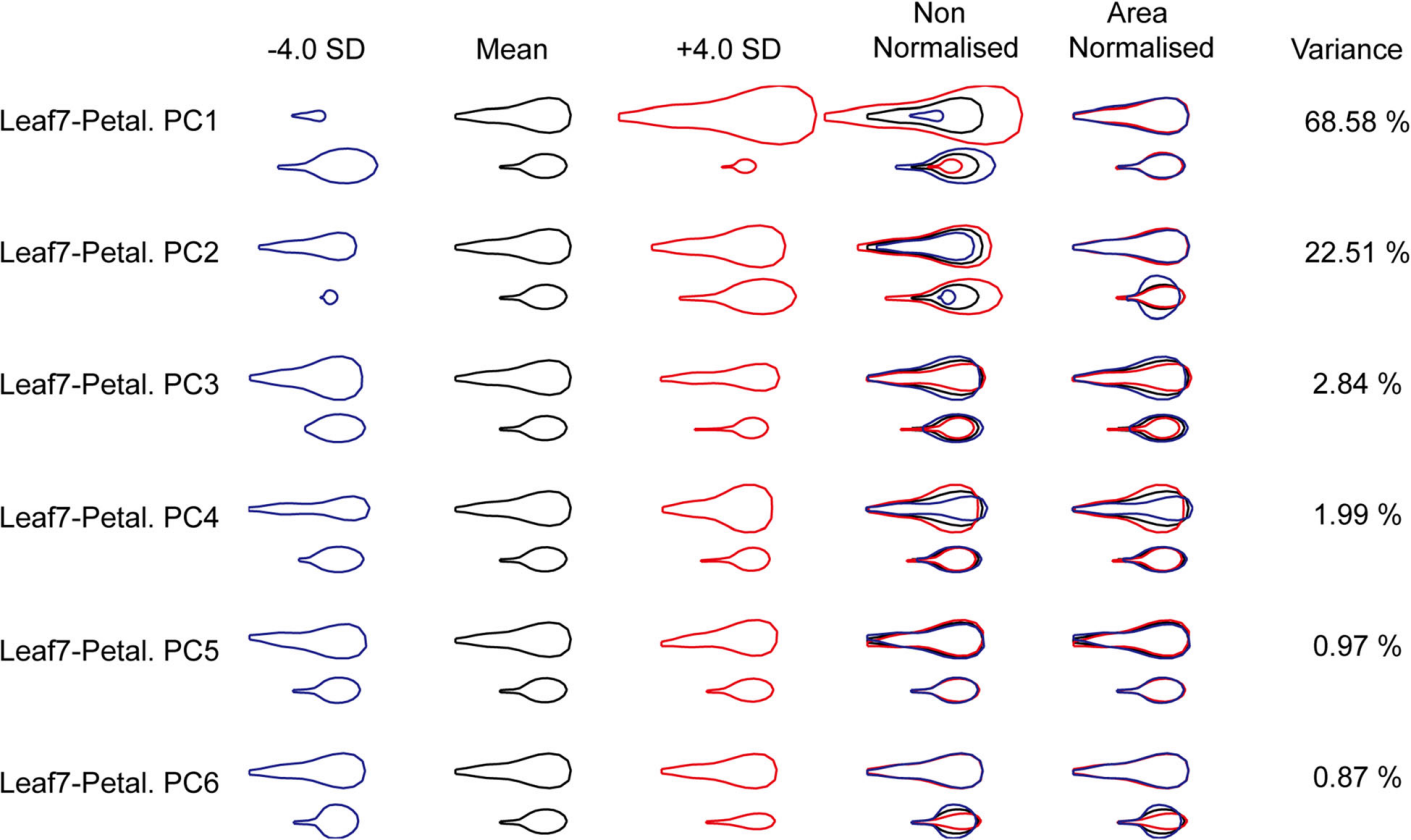
An integrated organ allometry model for the seventh leaves and petals. The effects on the leaf and petal outlines with decrease or increase in each PC by four standard deviations from the mean (black outlines) for all samples are represented by blue and red outlines respectively. The overlaid outlines illustrate the effects of each PC on organ shape and size covariation (area non-normalized) and organ shape covariation (area-normalized) separately. The proportion of the variance within the group captured by each PC to the total variance is given as a percentage

Similar to the Leaf4-Petal model, in the Leaf7-Petal model, PC1 accounted for 68.58% of the total variance, representing the negative size covariation between the seventh leaves and petals, whereas PC2 accounted for 22.51% of the total variance, representing the positive size covariation between the seventh leaves and the petals and the seventh leaf shape variance. PC3 accounted for 2.84% of the total variance, representing the positive shape (mainly in width) covariation, and PC4 accounted for 1.99% of the total variance, representing the negative shape (mainly in width) covariation. The other PCs represented only one organ shape or size variance, so they were not considered for further analysis (Fig. 4).

**Fig. 5:**
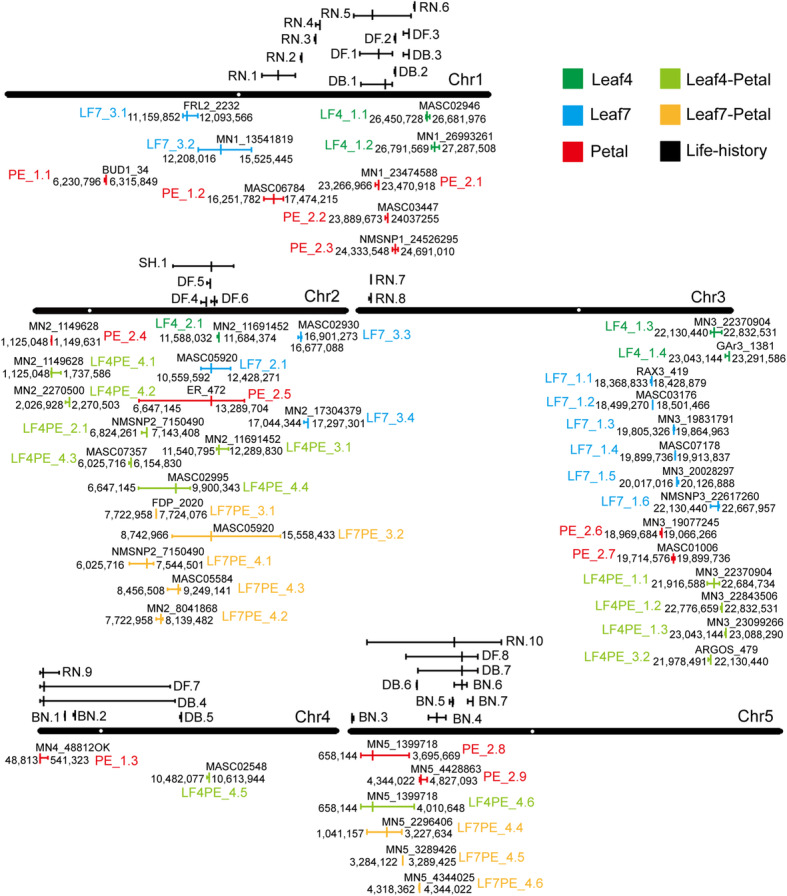
Chromosomal locations of QTLs for shape and size variation in Leaf4, Leaf7, petal, Leaf4-Petal, Leaf7-Petal and life history traits in MAGIC lines. The chromosomal location of identified QTLs is shown on the five *Arabidopsis* chromosomes. Each QTL is indicated by a line segment, with the extent of the line indicating the 95% confidence region and the peak SNP shown by a long vertical line in the QTL region. QTLs for different traits are shown in different colours: the QTLs for Leaf4 allometry are shown in dark green and named LF4 (LF4_1, Leaf4.PC1; LF4_2, Leaf4.PC2); the QTLs for Leaf7 allometry are shown in blue and named LF7 (LF7_1, Leaf7.PC1; LF7_2, Leaf7.PC2; LF7_3, Leaf7.PC3); the QTLs for petal allometry are shown in red and named PE (PE_1, Petal.PC1; PE_2, Petal.PC2); the QTLs for Leaf4-Petal shape and size covariation are shown in light green and named L4PE (L4PE_1, Leaf4-Petal.PC1; L4PE_2, Leaf4-Petal.PC2; L4PE_3, Leaf4-Petal.PC3; L4PE_4, Leaf4-Petal.PC4); the QTLs for Leaf7-Petal shape and size covariation are shown in orange and named L7PE (L7PE_3, Leaf7-Petal.PC3; L7PE_4, Leaf7-Petal.PC4); the QTLs for life-history traits are all shown in the dark (BN, branch number; DB, days to bolting; DF, days to flower; RN, rosette leaf number; SH, stem height)

After QTL mapping in the MAGIC lines for the Leaf7-Petal model, two significant QTLs for PC3 and six significant QTLs for PC4 were identified, whereas no significant QTL was identified in PC1 and PC2 (Fig. 5, Supplementary Table 5, Supplementary Figure 8). Moreover, candidate genes were also identified (Supplementary Tables 6 and 7). The QTL LF7PE_3.2 in PC3, which represented the positive shape (mainly in width) covariation between the seventh leaves and petals, had the most rounded leaves and petals in *Ler-0* and the narrowest leaves and petals in the *No-0* accession. In the 95% confidence region, there were 34 genes conferring alleles unique to the *Ler-0* or *No-0* accession. Among these genes, the GRF gene *AT2G22840*, pentatricopeptide repeat protein SLOW GROWTH1 (SLO1), ORGAN BOUNDARY1 (OBO1) and OVATE family of protein OFP16 have been reported to affect organ shape or size [25, 27–29]. Furthermore, the cyclin-dependent kinase inhibitor KRP4 [30] and the serine/threonine-protein kinase PINOID (PID) are involved in the regulation of auxin signalling [31]. *Growth-regulating factor 3* (*GRF3*), which regulates cell expansion in leaf and cotyledon tissues [28], as well as other genes associated with cell differentiation, cell expansion, cell wall modification, and flower time control genes, were also identified. The QTL LF7PE_4.4 in PC4, which represented the negative shape (mainly in width) covariation, had the narrowest leaves with the longest petioles and the most rounded petals in the *Po-0* accession. There were three genes with alleles unique to the *Po-0* accession, including *DME*, a transcriptional activator involved in gene imprinting; *peroxidase 2*, which influences cell elongation [32]; and *CYP712A2*, a member of CYP712A.

## Discussion

In this study, we defined a genetically controlled allometric space that captured most of the variation in leaf and petal shape and size among MAGIC lines. Among the loci identified, with the exception of the ER locus, the other QTLs were not identical to previously identified shape- and size-associated loci. Additionally, in these QTL confidence regions, many cell proliferation- and cell expansion-associated genes were isolated with unique alleles according to the accession distribution. Furthermore, we checked the candidate gene expression data and compared their promoter sequences in the 19 founder accessions of MAGIC lines and found that two candidate genes, *ERECTA* (*AT2G26330*) and *AGO4* (*AT2G27040*), in the ER locus on chromosome 2 had specific variations in promoter sequences unique to the maximal effects of accession. In addition, the specific promoter sequence variation also changed the expression level significantly (Supplementary Figure 11). However, more work is needed to test these loci, such as by constructing a NIL population.

### The interaction of leaf and petal shape and size variation

Interestingly, when we augmented the leaf and petal data to investigate the leaf and petal allometry covariation, the results showed that negatively correlated changes in leaf and petal size provided the largest component of allometric variation among MAGIC lines. The QTLs on chromosome 3 for Leaf4-Petal.PC1 overlapped with QTLs for Leaf4.PC1 with the smallest leaf size and the largest petal size in the *Can-0* accession allele. This indicated that the locus positively regulating fourth leaf size also negatively regulated petal size. The QTL on chromosome 2 for Leaf4-Petal.PC3 overlapped with Leaf4.PC2 and Petal.PC2 with the widest leaf and petal in the *Ler-0* accession allele, which indicated that the locus positively regulated both the leaf and petal width. The QTL on chromosome 2 for Leaf4-Petal.PC4 was identical to the QTL for Petal.PC2 with the same accession allelic effects distribution. Moreover, the QTL on chromosome 5 was identical to the QTL for Petal.PC2 with uncorrelated allelic effects distribution. In Leaf7-Petal.PC4, the QTL on chromosome 2 was identical to the QTL for Leaf7.PC2 and overlapped with the ER locus for Petal.PC2 with the maximum allelic effects in the *Ler-0* accession. Two QTLs on chromosome 5 for Leaf7-Petal.PC4 overlapped with the QTL for Petal.PC2, and the overlapping QTLs LF7PE_4.6 and PE_2.9 both obtained the minimum values in the *Ws-2* accession. In addition to these overlapping QTLs with leaf or petal allometry, others did not overlap and may have been independent loci for leaf and petal covariation.

### Leaf and petal allometry coordinated with local adaption

Additionally, life-history traits, such as days to bolting, days to flower, rosette leaf number, branch number, and stem height, were also measured in the MAGIC lines. As many significant phenotype correlations were found (Fig. 2), there were overlapping QTLs that could be used to explain the genetic correlation. After QTL mapping for these life-history traits (Fig. 5, Supplementary Tables 8 and 9, Supplementary Figure 9), the QTLs for the life-history traits and for the leaf and petal allometry model were compared (Supplementary Figure 10). In Leaf4.PC1, QTL LF4_1.1 overlapped with one linked QTL, RN.6, for rosette leaf number and showed the same allelic effects distribution with the maximum value in the *Po-0* accession. In Leaf7.PC2, QTL LF7_2.1 on chromosome 2 (~ 11.2 Mb) overlapped with three linked QTLs (DF.4, DF.5, and DF.6) for days to flower, with the highest value found in the *Bur-0* accession allele. In Petal.PC1, two QTLs, PE_1.2 on chromosome 1 (~ 16.9 Mb) and PE_1.3 on chromosome 4 (~ 0.05 Mb), overlapped with the QTLs RN.1 and RN.9 separately for rosette leaf number with uncorrelated allelic effects distribution. In Petal.PC2, there were four QTLs overlapping with the QTLs for days to bolting. Among these QTLs, the QTLs PE_2.2 and PE_2.3 on chromosome 1 overlapped with QTL DB.1, with the maximum value in the *Po-0* accession allele. Moreover, PE_2.9 on chromosome 5 overlapped with DB.6, with a maximum value in the *Can-0* accession allele. Others showed uncorrelated allelic effects distribution. There were also four QTLs for Petal.PC2 that overlapped with the QTLs for days to flower, and all showed uncorrelated allelic effects distribution. The overlapping QTLs for leaf and petal allometry with life-history traits provided a genetic basis in the correlation analysis. This colocalization may have resulted from pleiotropy or tightly linked causal genes, which indicated genetic integration among all traits.

## Conclusions

This is the first report on the genetic basis of allometry variation of leaves and petals and their interaction under the incorporated framework by using MAGIC lines. PCA for the MAGIC lines indicated that size variation was a major component of allometry variation and revealed negatively correlated changes in leaf and petal size. In this study, five QTLs for the fourth leaf, 11 QTLs for the seventh leaf, and 12 QTLs for petal size and shape were identified. These QTLs were not identical to those previously identified, with the exception of the ER locus. This indicated that the allometry variation was not simply the combination of organ width, length, and size. In addition, after QTL analysis of the leaf and petal integrated model, 12 QTLs were identified in association with the fourth leaf and petal allometry covariation, and eight QTLs were identified to be associated with the seventh leaf and petal allometry covariation. The QTL overlap explained the allometry correlation within different leaves and the homologous organs leaf and petal. However, some specific QTLs between Leaf4 and Leaf7 may explain the leaf allometry divergence, which may be associated with leaf developmental constraints. Additionally, the correlation of life history traits with leaf and petal allometry and the QTL overlap hinted at the genetic integration and the interaction of organ allometry with local adaptation.

## Methods

### Plant material and growth conditions

The large population of 527 RILs [14] was obtained from the University of Oxford in the United Kingdom and then propagated at Shandong Normal University in China. Seeds were sterilized for 10 min in 75% ethanol, washed in 95% ethanol four to six times, and then suspended in 0.1% agar. All lines were grown separately in 1/2 Murashige and Skoog medium. The seeds were subsequently grown under the following conditions in a plant incubator (Percival Scientific, Inc): 22 °C/18 °C (Day/Night) and a long photoperiod of 16 h/8 h (Day/Night) after treatment for 4 days at 4 °C in the dark for stratification. After they were grown in the medium for 7 days, the seedlings were transplanted into soil when the true leaves could be seen. For each line, we planted eight seedlings, with four seedlings per pot, which were randomly assigned to a tray. The trays were rotated throughout the incubator every week.

### Leaf and petal collection

After the first flower of the plant had opened, the fourth and seventh leaves of each plant were picked, flattened, and then glued onto paper for scanning to record the leaf shape. In total, the fourth leaves were obtained from 232 lines, and the seventh leaves were obtained from 215 lines (Supplementary Table 1). Because the leaves are more susceptible to environmental influences during growth, we calculated the average area of all obtained leaves in the same line and retained leaves with a difference in the range of +/− 20% for further analysis. To measure the shape and size of the petals, we picked and dissected the floral buds using a stereomicroscope at fully reflexed petal stage 13 when the buds had fully opened and the petals were visible and in anthesis. All four petals, four sepals, six stamens, and one pistil were removed, placed on 1% agar on a plate, and photographed with a Leica camera. Only buds between bud positions 4 and 10 on the main stem were used. Two flowers were dissected per plant, and each line we collected had at least four plants. Since some of these lines did not bloom properly under current planting conditions, we finally obtained petals from 345 lines for model construction (Supplementary Table 1).

### Modelling in leaves and petals

After images from all of the lines of the fourth and seventh leaves and the petals were prepared, the digital images were properly oriented (with the tip always pointing to the right and with good horizontality) using Photoshop CS5 software (Adobe Inc.). After that, we used MATLAB R2007b software (MathWorks Inc.) and the AAM Toolbox (version 6.5) [10] to construct the model of each individual leaf and petal separately [1]. The outline of each leaf and petal was represented by the Cartesian coordinates of its 25 points, which were placed around the leaf and petal outline using the leaf (Le) and petal (Pe) templates. These points were plotted to show the pattern of allometry in the data set, and PCA was used on the whole data set to identify trends in variation.

### Statistical analyses

Broad-sense heritability (*H*^*2*^) was estimated for each trait as in Gnan et al. [33], represented by the ratio of the variance among lines (approximately eight plants for each line) to the total variance. Pairwise Pearson correlations were calculated with line means between the traits measured by using PASW Statistics 18 software (SPSS Inc.). QTL analyses were then performed using the R software package HAPPY [14] for the Leaf4 PCs, Leaf7 PCs, petal PCs, argument of Leaf4-Petal PCs, argument of Leaf7-Petal PCs, and life-history traits. Two QTLs located less than 1 Mb apart were considered as overlapping QTLs reflecting genetic pleiotropy [33].

## Supplementary Information

**Additional file 1: Figure S1.** PCA was applied to the Leaf4, Leaf7, and petal data sets to identify trends in shape and size variations among MAGIC lines. The outline of a leaf was represented by the Cartesian coordinates of its 25 points, and similarly, a petal was represented by 25 points in the same way. The GIF showed the data set for each PC expressed in standard deviations from the mean position of the point within the collection of leaves and petals. **Figure S2.** Range of PC values obtained for the leaf and petal allometric model. The mean of each MAGIC line is represented by a blue diamond, and the bars display the range of all values observed for this line. PC units are standard deviations. **Figure S3.** Variations along each PC for Leaf4, Leaf7 and Petal allometry within the MAGIC lines. Each histogram represents the distribution of MAGIC lines along one of the PCs from the leaf and petal allometry model. **Figure S4.** QTL scan of the PCAs for the fourth leaf allometry among MAGIC lines. **Figure S5.** QTL scan of the PCAs for the seventh leaf allometry among MAGIC lines. **Figure S6.** QTL scan of the PCAs for petal allometry among MAGIC lines. **Figure S7.** QTL scan of the PCAs for the fourth leaf and petal allometry covariation among MAGIC lines. **Figure S8.** QTL scan of the PCAs for the seventh leaf and petal allometry covariation among MAGIC lines. **Figure S9.** QTL scan of the life-history traits among MAGIC lines. **Figure S10.** The genetic correlation between life-history traits and the allometry model. The vertical lines represent the QTLs overlapping with the peak SNP within 1 Mb. The different QTLs with the same accession allele conferring maximum effects are shown in red. **Figure S11.** The promoter sequence alignment and expression analysis of candidate genes. The candidate gene *ERECTA* (*AT2G26330*) in the LF7_2.1 locus, obtained the minimum Leaf7.PC2 value in the *Bur-0* accession, had specific variation in the *Bur-0* promoter sequence and had the lowest expression level in *Bur-0*. The second candidate gene *AGO4* (*AT2G27040*) in the LF7_2.1, PE_2.5 and LF7PE_3.2 loci had specific variation in the promoter in the *Ler-0* accession, with the maximum Leaf7.PC2, Petal.PC2 value and minimum Leaf7-Petal.PC3 value and obtained the highest expression level in *Ler-0*.

**Additional file 2: Table S1.** The phenotype data for all traits used for QTL analysis.

**Additional file 3: Table S2.** Significant QTL detected for the leaf and petal allometry models.

**Additional file 4: Table S3.** The estimated value for each of the 19 parental alleles at each detected QTL for leaf and petal allometry models.

**Additional file 5: Table S4.** The candidate genes account for leaf and petal allometry variation.

**Additional file 6: Table S5.** Significant QTL detected for the leaf and petal allometry covariation.

**Additional file 7: Table S6.** The estimated value for each of the 19 parental alleles at each detected QTL for leaf and petal allometry covariation.

**Additional file 8: Table S7.** The candidate genes account for leaf and petal allometry covariation.

**Additional file 9: Table S8.** The significant QTL detected for life-history traits in MAGIC lines.

**Additional file 10: Table S9.** The estimated value for each of the 19 parental alleles at each detected QTL for life-history traits.

## Data Availability

All data generated or analyzed during this study are included in this published article and its additional files. The raw data used for this research and the supplementary material are available in Figshare (https://figshare.com/s/90c637df9f8965f346c8). Dataset 1, which is used for PCA, contains all the cropped images and point models for each plant of the MAGIC lines. The values of each PC and the life history traits for each plant in the MAGIC lines are listed in Dataset 2. Dataset 3 is the record of correspondence between planting ID and the MAGIC line ID. File 1 contains the R source code files for QTL mapping in MAGIC lines.

## Abbreviations

MAGIC: Multiparent advanced generation intercross
PCA: Principal component analysis
PCs: Principal components
QTL: Quantitative trait loci
