# Supplementary material for: The genetic control of leaf and petal allometric variations in Arabidopsis thaliana
Source: BMC Plant Biol. 2020 Dec 7;20:547. doi: 10.1186/s12870-020-02758-w (PMC7720488; doi:10.1186/s12870-020-02758-w)
Supplement: Supplementary file 1 — Additional file 1: Figure S1. PCA was applied to the Leaf4, Leaf7, and petal data sets to identify trends in shape and size variations among MAGIC lines. The outline of a leaf was represented by the Cartesian coordinates of its 25 points, and similarly, a petal was represented by 25 points in the same way. The GIF showed the data set for each PC expressed in standard deviations from the mean position of the point within the collection of leaves and petals. Figure S2. Range of PC values obtained for the leaf and petal allometric model. The mean of each MAGIC line is represented by a blue diamond, and the bars display the range of all values observed for this line. PC units are standard deviations. Figure S3. Variations along each PC for Leaf4, Leaf7 and Petal allometry within the MAGIC lines. Each histogram represents the distribution of MAGIC lines along one of the PCs from the leaf and petal allometry model. Figure S4. QTL scan of the PCAs for the fourth leaf allometry among MAGIC lines. Figure S5. QTL scan of the PCAs for the seventh leaf allometry among MAGIC lines. Figure S6. QTL scan of the PCAs for petal allometry among MAGIC lines. Figure S7. QTL scan of the PCAs for the fourth leaf and petal allometry covariation among MAGIC lines. Figure S8. QTL scan of the PCAs for the seventh leaf and petal allometry covariation among MAGIC lines. Figure S9. QTL scan of the life-history traits among MAGIC lines. Figure S10. The genetic correlation between life-history traits and the allometry model. The vertical lines represent the QTLs overlapping with the peak SNP within 1 Mb. The different QTLs with the same accession allele conferring maximum effects are shown in red. Figure S11. The promoter sequence alignment and expression analysis of candidate genes. The candidate gene ERECTA (AT2G26330) in the LF7_2.1 locus, obtained the minimum Leaf7.PC2 value in the Bur-0 accession, had specific variation in the Bur-0 promoter sequence and had the lowest expression level in Bur [file 12870_2020_2758_MOESM1_ESM.pptx]

## Slide 1
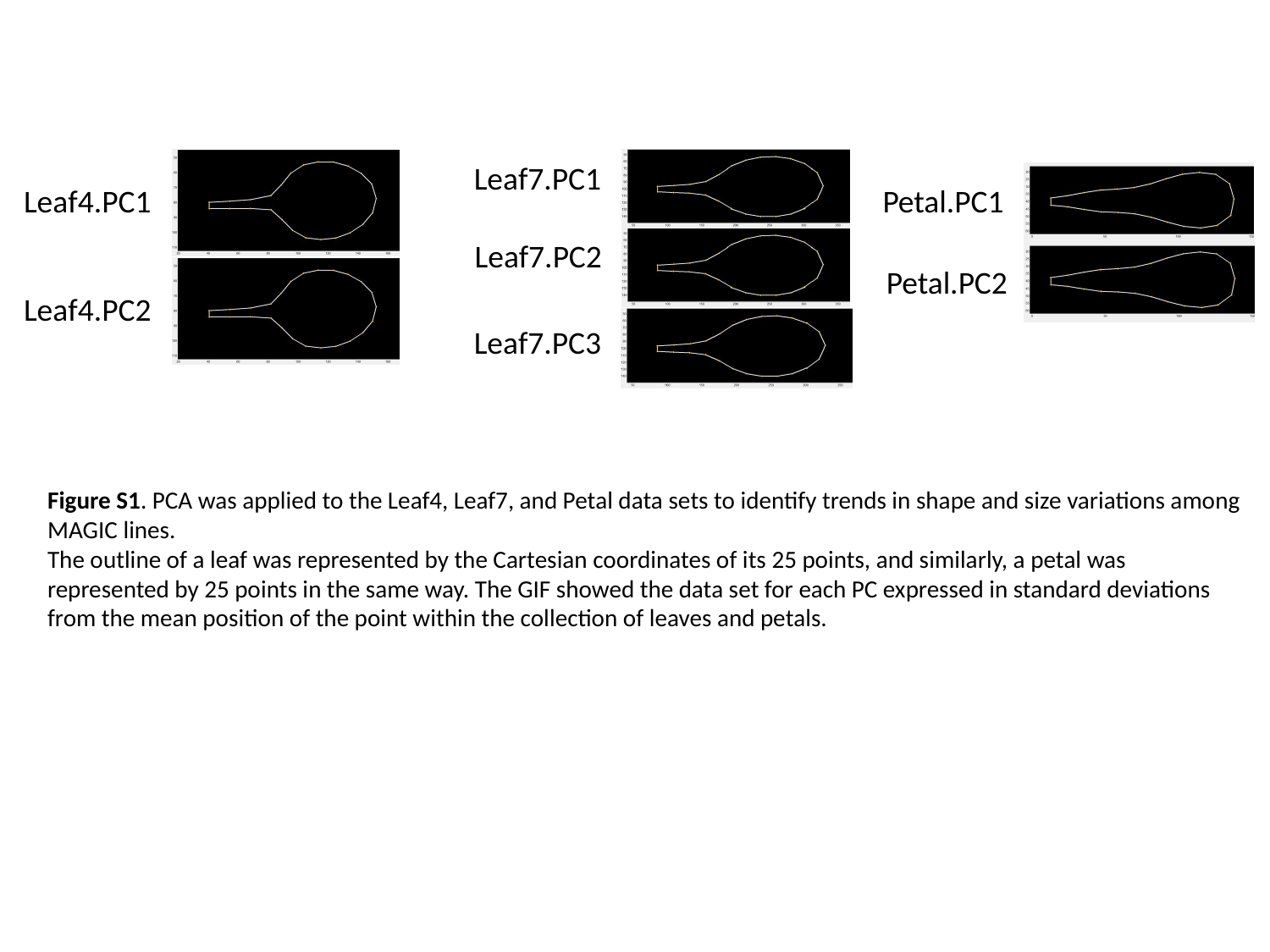

Leaf7.PC1
Leaf4.PC1
Petal.PC1
Leaf7.PC2
Petal.PC2
Leaf4.PC2
Leaf7.PC3
Figure S1. PCA was applied to the Leaf4, Leaf7, and Petal data sets to identify trends in shape and size variations among MAGIC lines.
The outline of a leaf was represented by the Cartesian coordinates of its 25 points, and similarly, a petal was represented by 25 points in the same way. The GIF showed the data set for each PC expressed in standard deviations from the mean position of the point within the collection of leaves and petals.

## Slide 2
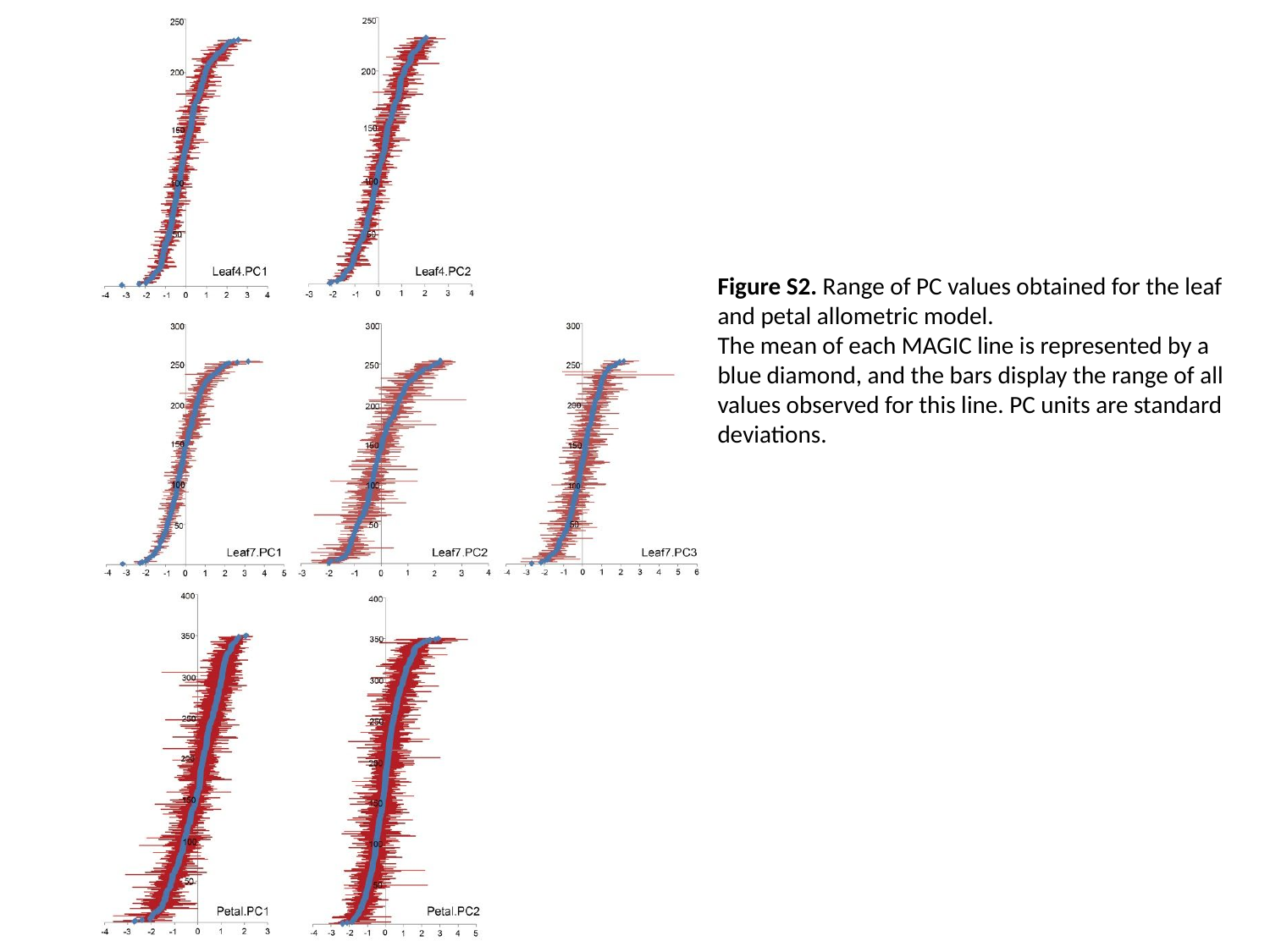

Figure S2. Range of PC values obtained for the leaf and petal allometric model.
The mean of each MAGIC line is represented by a blue diamond, and the bars display the range of all values observed for this line. PC units are standard deviations.

## Slide 3
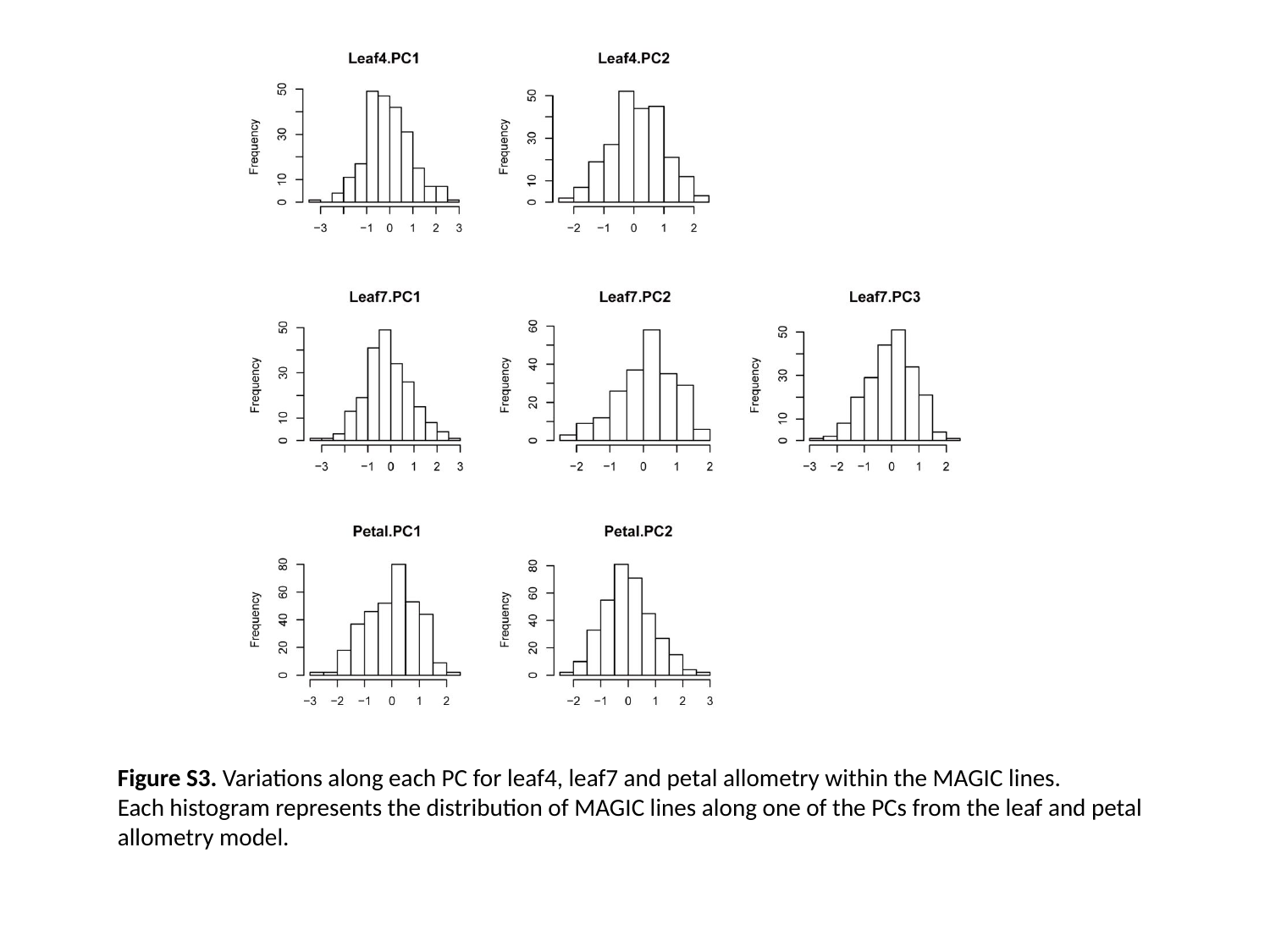

Figure S3. Variations along each PC for leaf4, leaf7 and petal allometry within the MAGIC lines.
Each histogram represents the distribution of MAGIC lines along one of the PCs from the leaf and petal allometry model.

## Slide 4
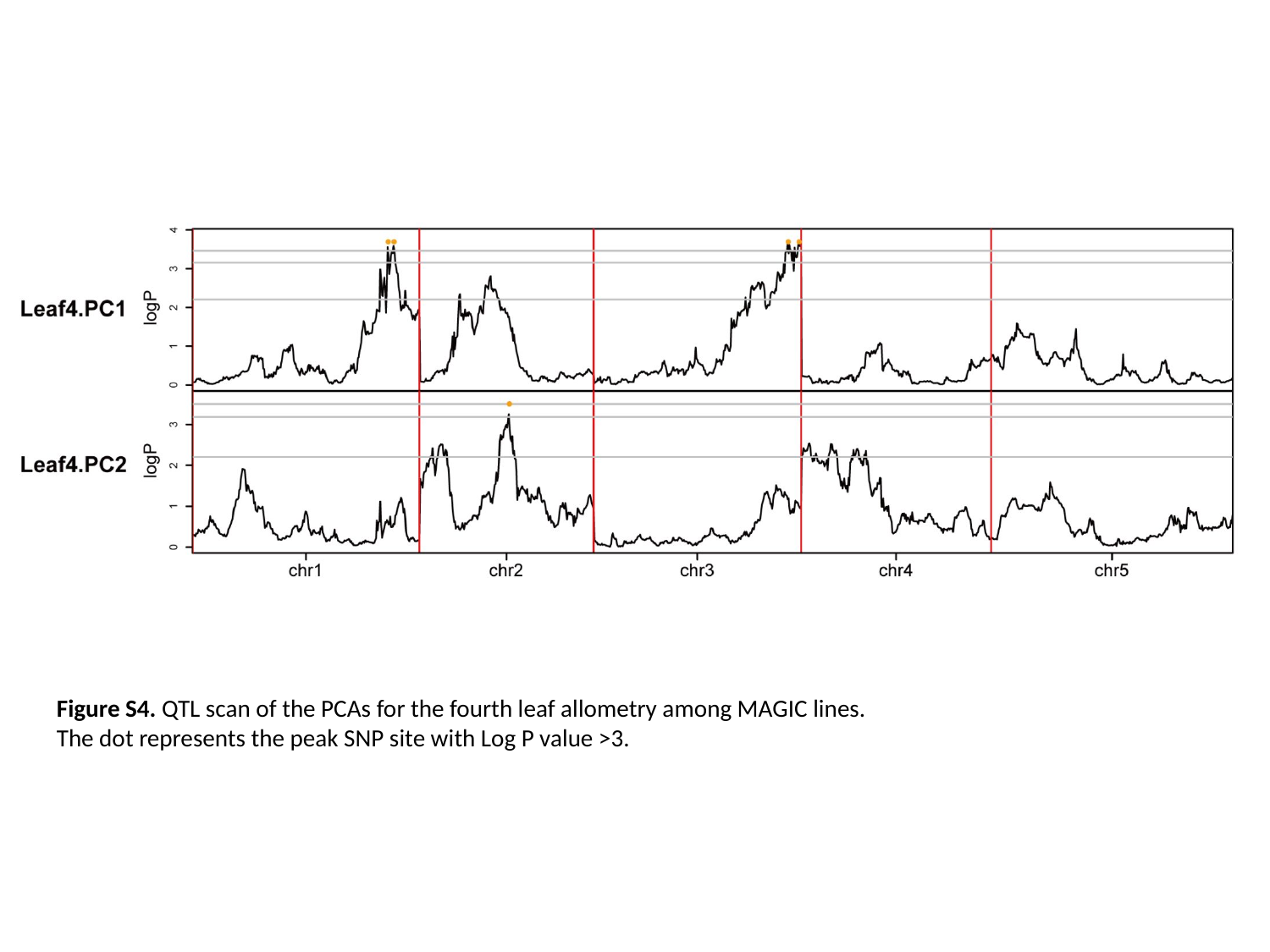

Figure S4. QTL scan of the PCAs for the fourth leaf allometry among MAGIC lines.
The dot represents the peak SNP site with Log P value >3.

## Slide 5
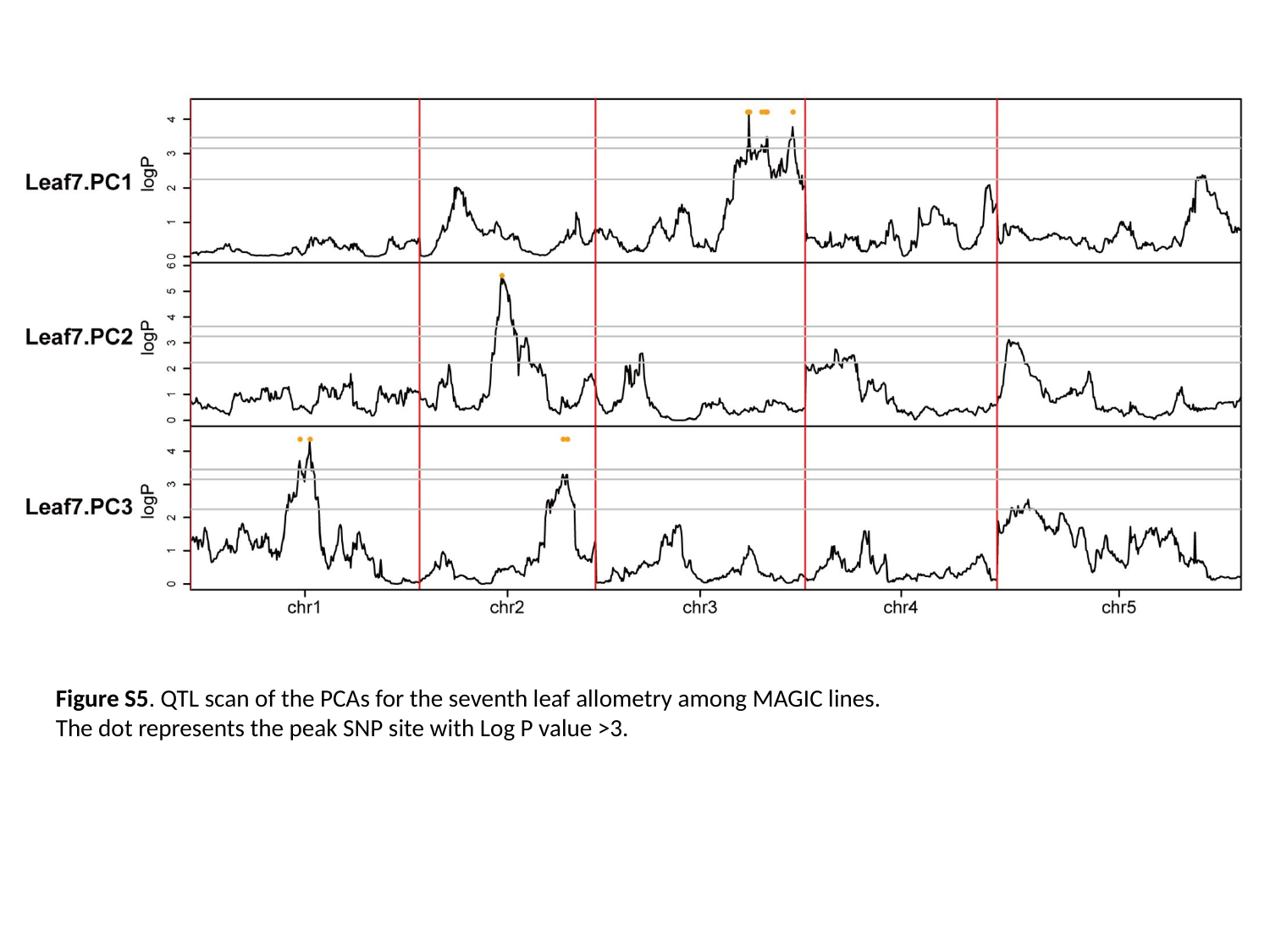

Figure S5. QTL scan of the PCAs for the seventh leaf allometry among MAGIC lines.
The dot represents the peak SNP site with Log P value >3.

## Slide 6
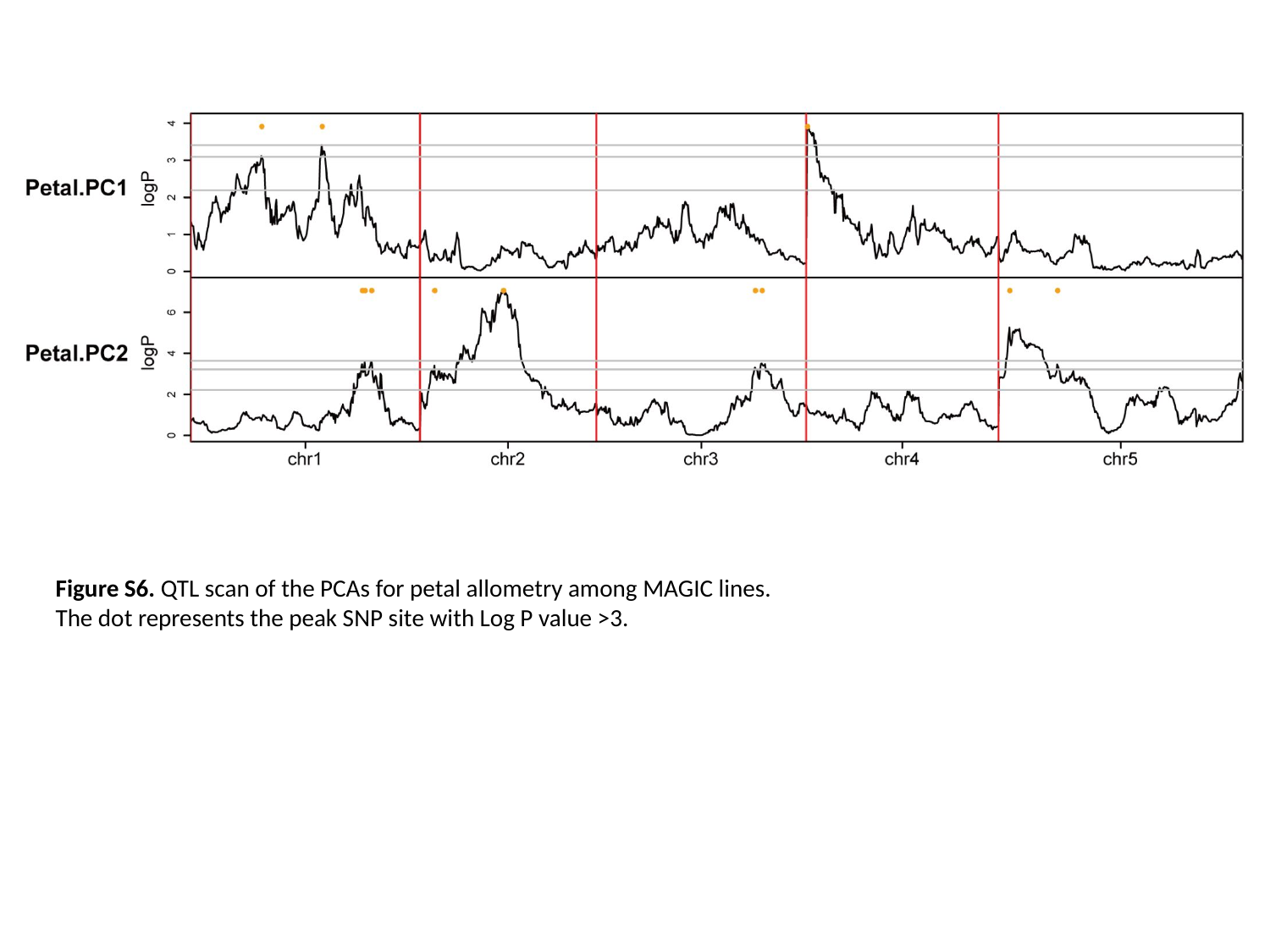

Figure S6. QTL scan of the PCAs for petal allometry among MAGIC lines.
The dot represents the peak SNP site with Log P value >3.

## Slide 7
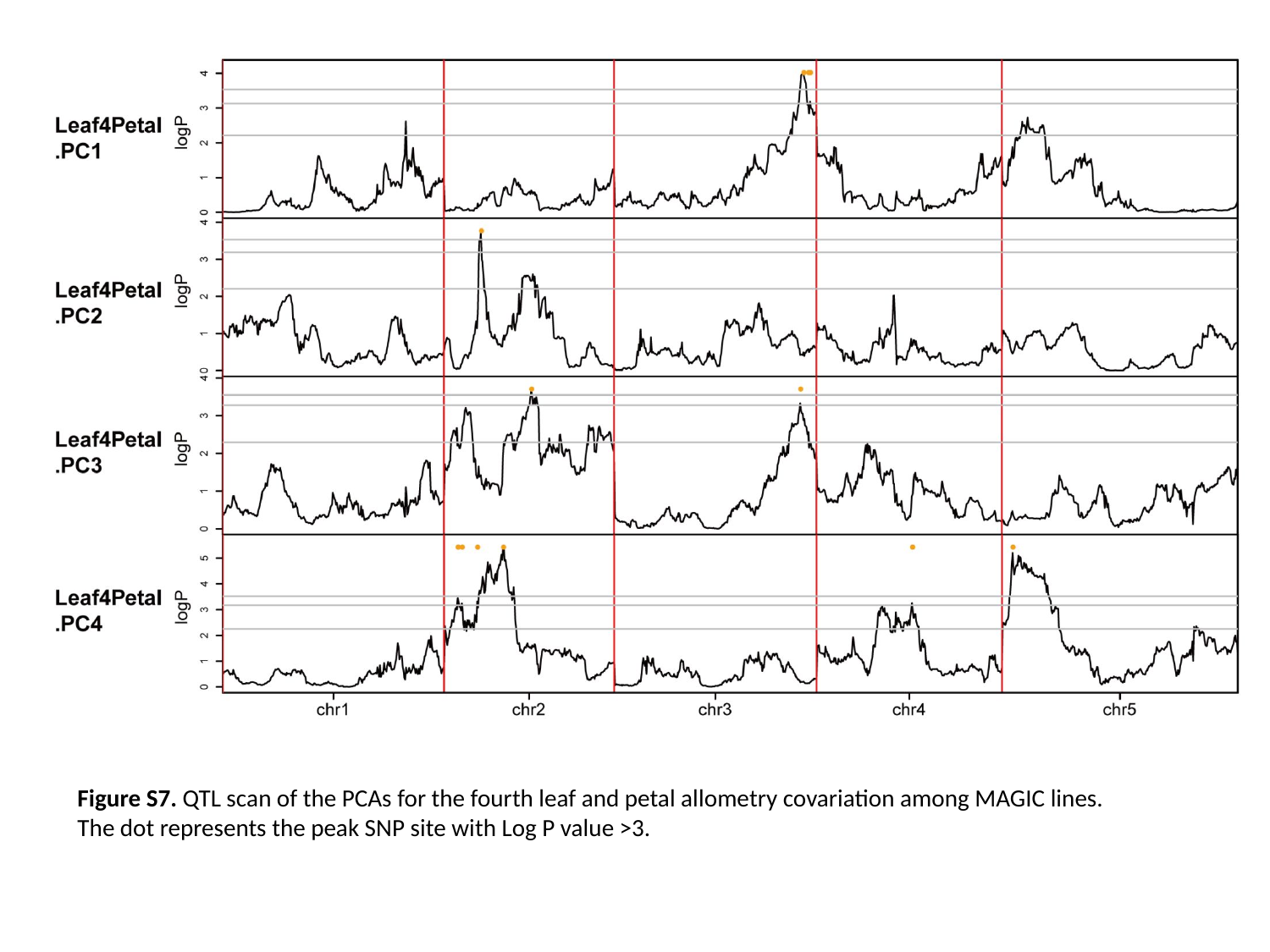

Figure S7. QTL scan of the PCAs for the fourth leaf and petal allometry covariation among MAGIC lines.
The dot represents the peak SNP site with Log P value >3.

## Slide 8
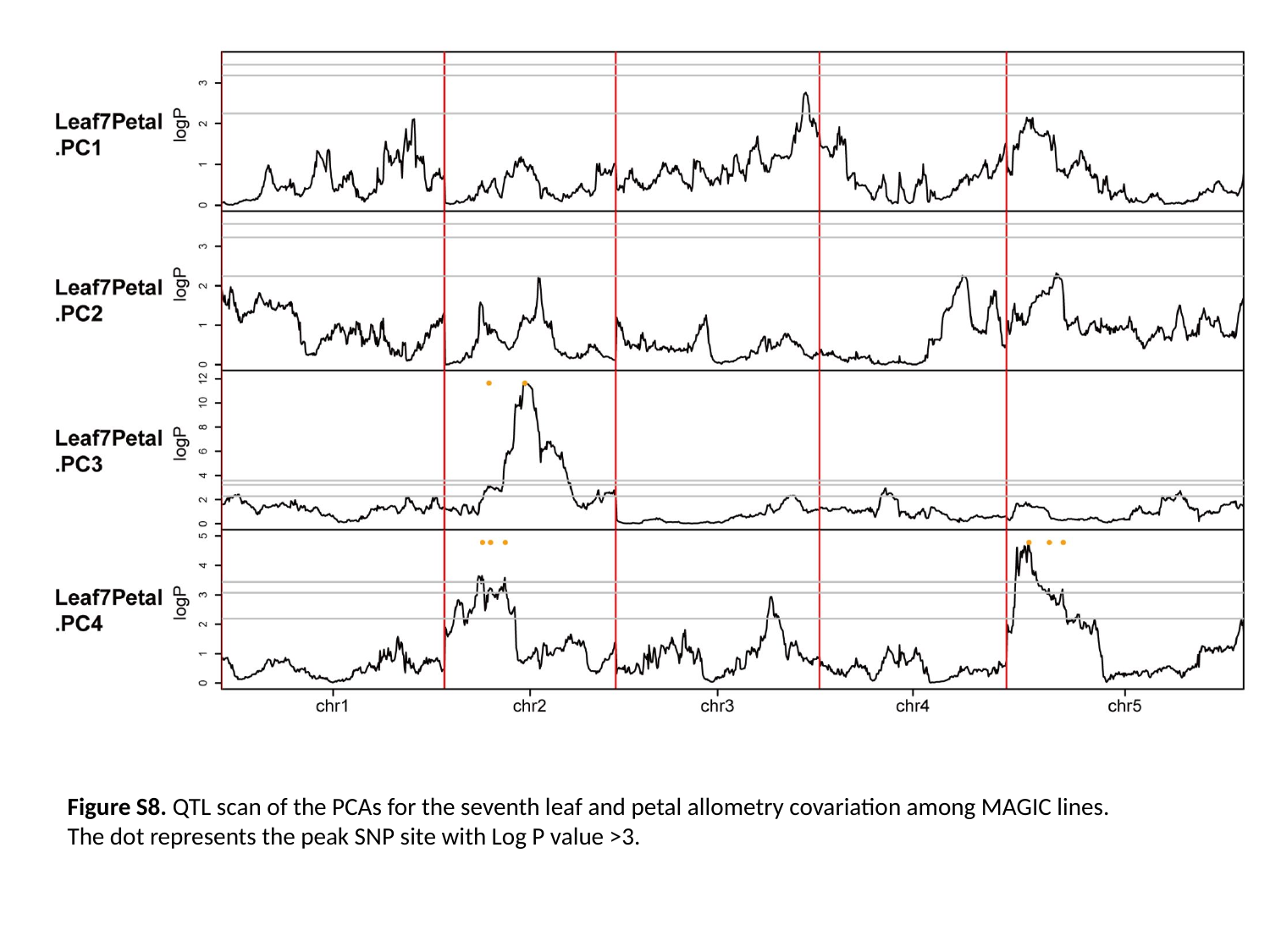

Figure S8. QTL scan of the PCAs for the seventh leaf and petal allometry covariation among MAGIC lines.
The dot represents the peak SNP site with Log P value >3.

## Slide 9
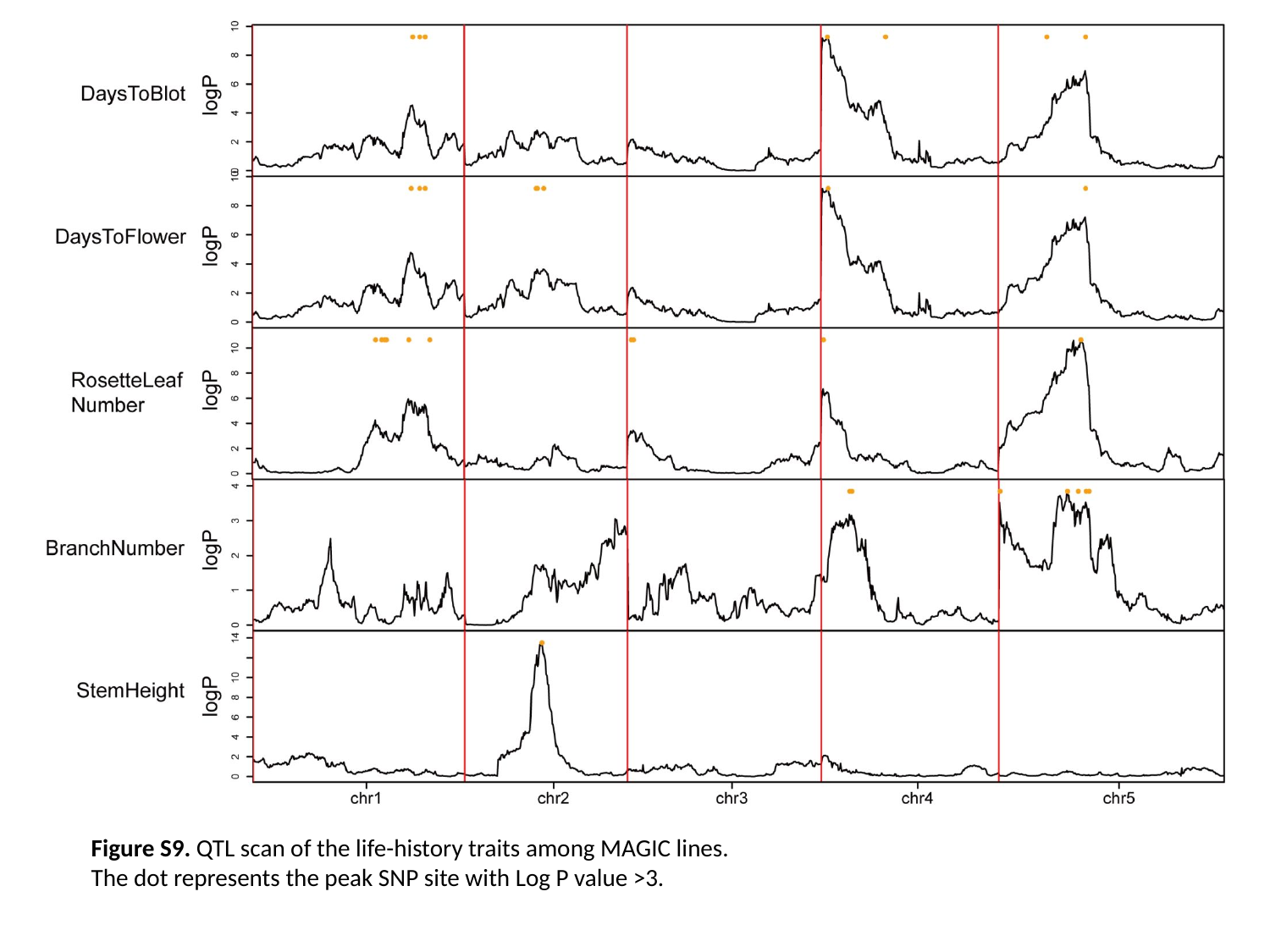

Figure S9. QTL scan of the life-history traits among MAGIC lines.
The dot represents the peak SNP site with Log P value >3.

## Slide 10
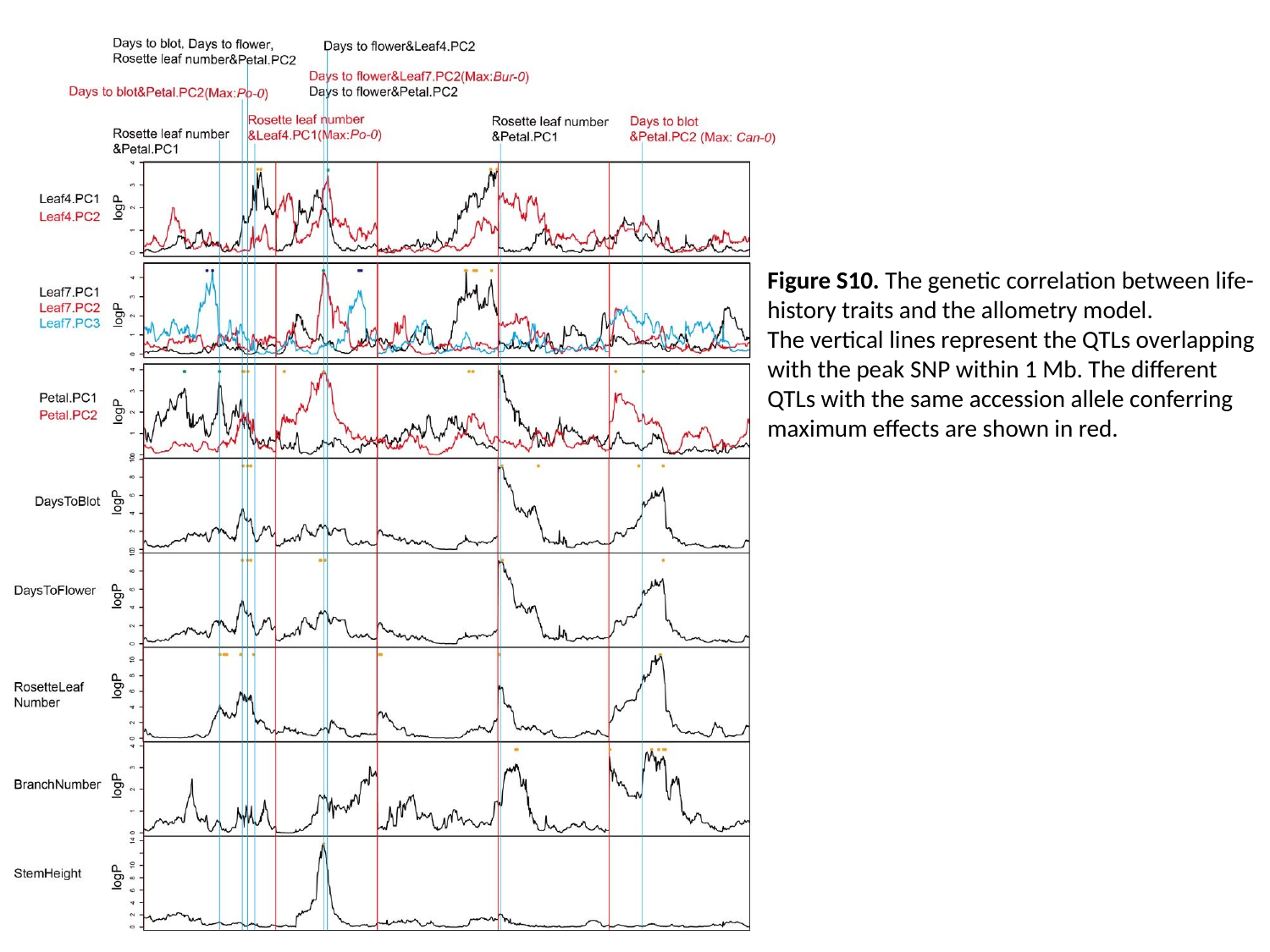

Figure S10. The genetic correlation between life-history traits and the allometry model.
The vertical lines represent the QTLs overlapping with the peak SNP within 1 Mb. The different QTLs with the same accession allele conferring maximum effects are shown in red.

## Slide 11
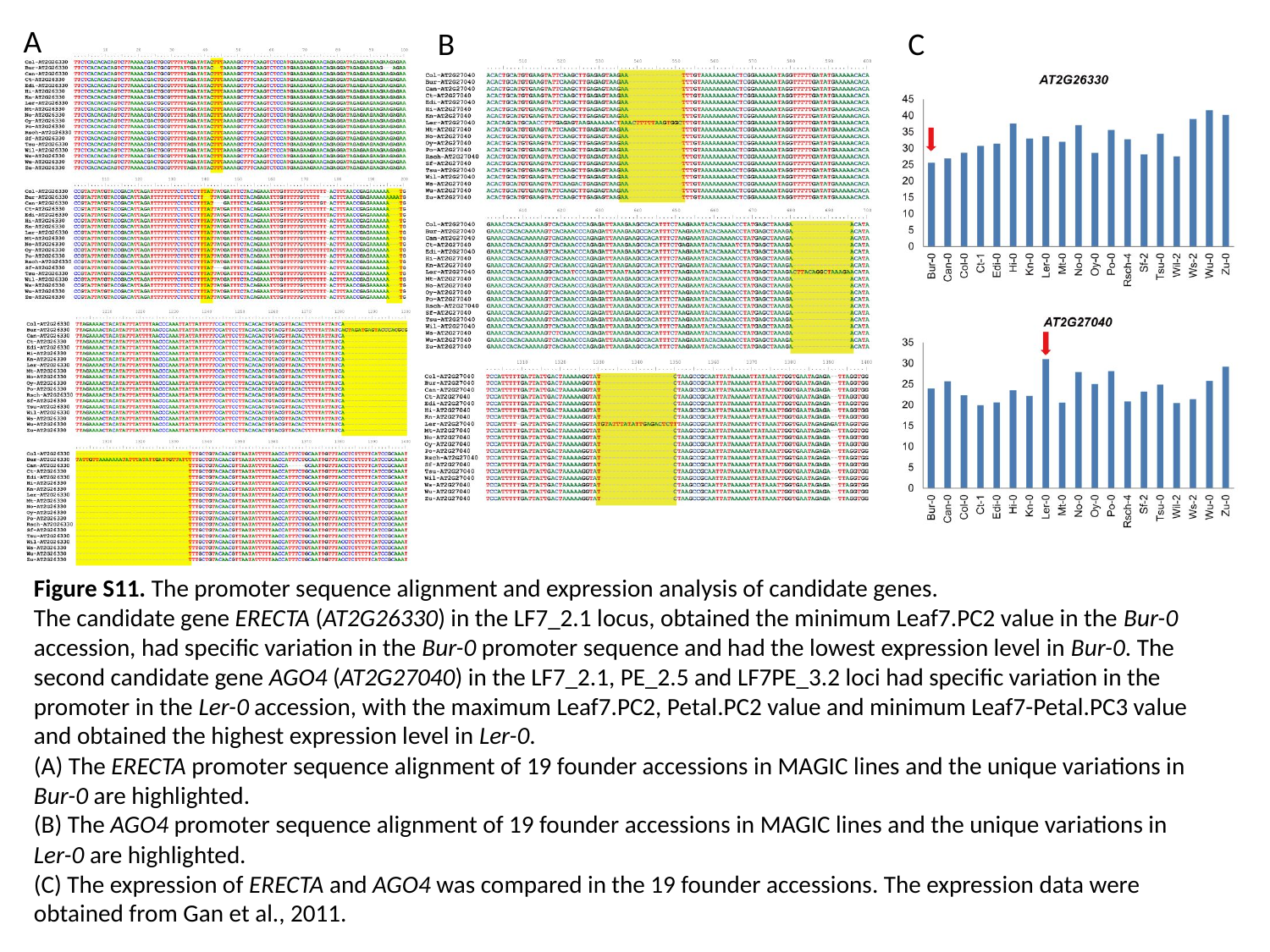

A
B
C
Figure S11. The promoter sequence alignment and expression analysis of candidate genes.
The candidate gene ERECTA (AT2G26330) in the LF7_2.1 locus, obtained the minimum Leaf7.PC2 value in the Bur-0 accession, had specific variation in the Bur-0 promoter sequence and had the lowest expression level in Bur-0. The second candidate gene AGO4 (AT2G27040) in the LF7_2.1, PE_2.5 and LF7PE_3.2 loci had specific variation in the promoter in the Ler-0 accession, with the maximum Leaf7.PC2, Petal.PC2 value and minimum Leaf7-Petal.PC3 value and obtained the highest expression level in Ler-0.
(A) The ERECTA promoter sequence alignment of 19 founder accessions in MAGIC lines and the unique variations in Bur-0 are highlighted.
(B) The AGO4 promoter sequence alignment of 19 founder accessions in MAGIC lines and the unique variations in Ler-0 are highlighted.
(C) The expression of ERECTA and AGO4 was compared in the 19 founder accessions. The expression data were obtained from Gan et al., 2011.
